# Book Reviews

**Published:** 1823-09

**Authors:** 


### CRITICAL ANALYSIS
OF
### ENGLISH AND FOREIGN LITERATURE.

---

### DIVISION I.

### ENGLISH.

ART. I.—*A Treatise on the Epidemic Puerperal Fever, as it prevailed in Edinburgh in* 1821-22. *To which is added an Appendix, containing the Essay of the late* Dr. GORDON *on the Puerperal Fever of Aberdeen, in* 1789-90-91-92. By WILLIAM CAMPBELL, M.D. Fellow of the Royal College of Surgeons, one of the Medical Officers of the Royal Public Dispensary, Lecturer on Midwifery, &c.—8vo. pp. 400. Edinburgh and London, 1822.

ART. II.—*A Treatise on the Disease termed Puerperal Fever; illustrated by numerous Cases and Dissections.* By JOHN MACKINTOSH, M.D.—8vo. pp. 323. Cadell, London, 1822.

ART. III.—*Report on Puerperal Fever, in Answer to Queries from the General Board of Health.* By JOHN DOUGLAS, M.D. Licentiate of the King and Queen's College of Physicians.—(*Dublin Hospital Reports,* vol. iii.)

COMPARISONS are odious; yet we cannot resist from declaring our opinion, on the very threshold of our present article, that the first of these works is by far the best and most ably written; and that, on the accuracy of the statements contained in it, we feel better disposed to place implicit reliance. Indeed, we may safely, and without doing injustice either to Dr. MACKINTOSH or to our readers, dispatch his treatise in a few pages.

In the first section, Dr. Mackintosh details the literary history of puerperal fever; consisting, first, in allusions to what the older physicians have written respecting the nature and treatment of this peculiar affection of childbed; secondly, in giving a sort of chronological recapitulation of the several epidemics of this disease, in France, England, Scotland, &c.; thirdly, in a repetition of the doctrines held forth, and the treatment proposed, by the more modern practitioners, including Drs. Leake, Hulme, William Hunter, Wallace, Johnson, Mackenzie, Walsh, Denman, Gordon, Clarke, Hamilton, jun. and Messrs. Hay, White, and Burns. The curious fact that the most eminent among these men have uniformly asserted the disease in question to be most fatal in its consequences, whatever be the treatment adopted, is duly recorded by Dr. Mackintosh, particularly as it regards the present distinguished professor of midwifery in the university of Edinburgh; but our author seems to have done this more with a view to contrast their assertions with what he himself boldly advances, in the sequel of his work, as the result of his own experience, than with the intention of showing in what those great men have particularly erred, when they candidly proclaimed the unsuccessful result of their efforts.

The contrasted positions of Dr. Mackintosh we will give in his own language, not very remarkable for that *caution* which should distinguish an experienced practitioner; but which it is, perhaps, too much to expect in a junior physician, full of ardour, and fond of general and sweeping conclusions.

"From what I have seen of this disease, and of others equally severe, which bear a strict analogy with it;, and from all that has been written on the subject, as well as from the success of the practice adopted by those with whom I have been led to agree; I think I am justified in asserting, that four-fifths of the patients in a well-regulated hospital ought to be cured; and, in private practice, I feel convinced that at least two-thirds should be restored to their families, notwithstanding the prejudices which are known to prevail among lying-in women and nurses, and the want of that proper degree of authority which a physician ought to possess over his patient."

Without stopping to inquire how Dr. Mackintosh, even in his bright arithmetical promises, calculated to a fractional nicety, (a precision hitherto unknown in prognosis,) could be induced to favour less patients in private than in public practice, in meting out their respective shares of consolation,* we shall proceed to look for the grounds of his assertion: nor shall we have to go far before we show that Dr. Mackintosh has completely failed to show his warranty, either from his success or the extent of his experience, for holding out such unqualified promises.

To the literary, succeeds the *medical* history of "the disease termed puerperal fever," in which we meet with the author's own ideas respecting the nature of that disease, and the analogy said to exist between it and peritoneal inflammation occurring in the non-puerperal state, on the one hand, and also between it and some other *congestive* and inflammatory affections of the viscera in the *thoracic* and abdominal cavities, on the other hand; particularly some varieties of dysentery, intermittent yellow fever, cholera morbus, and the berrebery. To which of these complaints the word thoracic is meant to apply, we are not told.

Our readers can probably guess from this statement that Dr. Mackintosh divides puerperal fever into two varieties; and they

* In well-regulated hospitals, according to our author, four-fifths ought to be cured,—that is to say, *twelve* in fifteen; but in private practice only two-thirds, or *ten* in fifteen. Is not this reversing the usual order of events in medical practice? What makes this statement the more singular, is Dr. Mackintosh's confession, at page 302, that he has not seen one case of the disease in hospital practice.

are right. These varieties are, first, the congestive puerperal fever; secondly, the inflammatory puerperal fever.

The CONGESTIVE VARIETY is thus delineated:

"In the first variety, there is a manifest oppression of the vital powers, commencing generally with a rigor, more or less severe; coldness of the surface, particularly of the extremities; paleness of the countenance, expression of anxiety in the features; a soft compressible pulse, which in some cases has a full beat, at others it is contracted; respiration sometimes laborious, at others amounting only to a slight difficulty. Pain in some part of the abdomen is complained of, for the most part over its whole surface; in some cases confined to the epigastric or hypochondriac regions. In this state of the system it is sub-acute, increased, however, upon strong pressure; or excessively acute, the patient dreading the slightest touch. The patient is quite sensible, although she speaks little; aware of her awful condition, she desponds. In some cases, diarrhœa precedes, or soon follows, these symptoms; and she dies, sometimes in six, sometimes in twenty-four hours, in spite of the exhibition of brandy, which it is so much the custom to rely on. This, it must be confessed, is a rare case: it does now and then, however, occur; and, after it has existed an hour or two, no treatment will prove effectual. Prevention here is the grand point, and a good attentive practitioner can almost always see where the disease is to be expected." (p. 30—31.)

Of this "peculiar and alarming variety," the symptomatology of which is so minutely described by the author, it will be naturally conjectured that he must have witnessed not a few instances, to enable him to ground well his nosological division. We have, however, looked in vain throughout the book for any evidence that such has been the case. He, indeed, tells us (p. 33,) that this variety of puerperal fever (the *congestive*) first came under his notice at Woolwich, in 1808, in the case of the wife of a soldier in the Royal Artillery, who was attended by Mr. Hicks, and which proved fatal; and he afterwards adds, that he had several opportunities, in the West Indies, of witnessing appearances nearly of the same kind as those he had seen on the examination of the body of that unfortunate patient. But, although a distinct promise is made to refer to *those opportunities* in the sequel of the work, we have not been able to trace any such reference; nor indeed, what would have been more correct and impressive, any detailed account of cases of the *congestive variety*, as having occurred to the author either at home or abroad. Whether such an omission be the result of oversight or otherwise, we will not undertake to assert.

Matters being thus disposed by the author himself, can it be expected of us that we shall adopt his first, or congestive, variety of the disease termed puerperal fever? Decidedly not; and for two reasons: first, because the author has failed to bring forward *any* positive evidence to prove the correctness of his views; and secondly, because, without such evidence, the *ipso dixit* of any physician, now-a-days, goes for nothing.

This being the case, we need not say a word of Dr. Mackintosh's etiology of this supposed variety of puerperal fever, and very little, indeed, of his treatment. Nor could we conscientiously recommend to our readers this part of the author's treatise as a guide to the study of one of the most obscure pathological affections of puerperal women. Dr. Mackintosh himself acknowledges, at pages 257 and 58, that his experience in the congestive variety of puerperal fever is limited, and that he can only treat of it by the analogies which it bears to other congestive diseases, and the similarity of the appearances on dissection. Upon that train of analogies, Dr. Mackintosh founds his treatment of the "congestive variety." Now, we ask, ought any disease to be treated, in our days, according to the supposed analogy it bears to others? Was it not incumbent on Dr. Mackintosh, if such was his opinion, to show, by a faithful narrative of the cases of congestive puerperal fever which he had been called to see, and of the appearances on dissection which he states to have witnessed, where and how, and to what extent, their pretended analogies existed? Having completely neglected to do so, Dr. Mackintosh cannot be surprised at our expressing an opinion respecting this first part of his treatise, which must go to the overthrowing of a structure under which Dr. Mackintosh has failed to place a proper foundation. Indeed, there is so much confusion in that part of Dr. Mackintosh's work on which we are descanting,—the treatment (by analogies) of the supposed congestive variety of puerperal fever,—that we cannot easily detect the real meaning of our author.

"This first variety of the disease," observes Dr. Mackintosh, (p. 257,) "is, fortunately for society, of rare occurrence;" and, "when it does take place, no treatment will have any avail, unless the symptoms are arrested in the very onset."—From these prefatory observations, one would naturally be led to suppose that the treatment recommended by the author *must have been of avail* in his hands, from the circumstance of it having been put into practice by him "in the very onset of the malady:" else how could he venture to recommend it? But no such thing. "In the cases which I have seen," Dr. Mackintosh proceeds to observe, "the time for remedy had gone by, and I was merely a silent witness to the speedy termination of life." How, then, can Dr. Mackintosh aver that the treatment he recommends will prove successful? As it is acknowledged, on the one hand, that the treatment in question has proved of no avail, when not adopted in the very onset of this variety of fever; and it is admitted, on the other, that in all the cases

(except one) which the author saw, it was too late to do good; it follows that Dr. Mackintosh has no reason for recommending his treatment. Moreover, which he, somewhat boastingly, tells us, (page 259,) "he has never had a case either of the congestive or of *the other form* of the disease," (but one,) when he has attended lying-in women from the first; what other conclusions, therefore, can we draw from all this information but these,—first, that the treatment recommended on the experience of our author has never been tried, (if it has been tried at all,) except under circumstances in which it could be *of no avail;* and therefore, secondly, that Dr. Mackintosh is not entitled to recommend, in such unqualified language as he has done, a treatment, of the value of which his own experience has not furnished the smallest particle of evidence.

Again: Dr. Mackintosh informed us, in another part of his book, page 123, "that, in the instances (of congestive puerperal fever) which fell under his notice in the West Indies, death soon followed." Of course, we are to understand that, in none of those cases, Dr. Mackintosh had attended the lying-in patients from the first, and that he had not seen the disease at its onset. If so, what becomes of his symptomatology of the disease, his etiology of it, and his treatment? Must they not, necessarily, be presumptive, in part at least, and not founded on actual observation? If Dr. Mackintosh, however, thought otherwise, and deemed himself warranted in all his conclusions and unqualified assertions on this subject, from the number of cases of the disease which he had attended or witnessed, why not detail at full length one, two, three, or more of them, (as, indeed, every author, anxious to establish a good ground for a new opinion respecting any disease, would have done,) if he really was in possession of the history of such cases? And, above all, why omit to state the appearances on dissection, that we might judge of the correctness of the alleged analogies between *his* variety of puerperal fever and other congestive diseases? With what ill grace must any excuse for neglecting to do so, come from the author who can launch forth into the following lamentation!

"I am informed that many cases of this variety of puerperal fever have occurred in this city at various periods; and it is deeply to be regretted that we have not had a hint of the appearances which were observed in the abdomen after death."

We think we need not go beyond this to show that, thus far, Dr. Mackintosh's performance is most feeble.

The second variety of the disease "termed puerperal fever," admitted by Dr. Mackintosh, is said to be less alarming, but not less fatal if neglected or overlooked for six hours. It is thus described:

"The attack comes on suddenly, from the first to the fifth day after delivery, the sooner the worse, and *vice versa.* It is ushered in with one or more rigors, or cold shiverings,—at one time severe and long-continued, at another slight and short in duration: in some instances there is no marked rigor, or sense of cold, which is however rare. This state is preceded, or immediately followed, by flushes of heat, pain, and increased action of the vascular system: this is usually termed re-action, or the state of excitement.

"The pain is fixed in some part of the abdomen, usually the hypogastric region, or at one or other side, where we shall find the uterine tumor, if we are called to the patient in an early stage of the disease, (because, *perhaps*, the peritoneum covering the uterus and the ovaria is, in general, the first part affected:) the pain, being sometimes slight at first, quickly becomes excruciating, so that the patient is soon unable to turn herself in bed; she lies upon her back, dreading the slightest touch; the weight of the bed-clothes, in some cases, becomes unsupportable. I think Dr. Hamilton takes notice that in all his cases the pain was very acute. From being for a short time confined to one spot, the pain extends over the whole cavity of the abdomen, or merely changes its place. In no case should the practitioner be satisfied with the woman's own account of this symptom; he should convince his mind fully, by pressing his hand over the abdomen: of course, he will do so with proper delicacy and gentleness. If there is any pain, pressure increases it to such a degree that, if her countenance is watched at the time, it cannot well be concealed. Young gentlemen must not think this a needless trouble. It is unaccountable how people sometimes deceive themselves, and try to impose on the medical attendants, from a dread that, should they complain, the use of the lancet, or the exhibition of a disagreeable medicine, will follow. It must be confessed that the pain is sometimes so slight, that the patient can bear a common degree of pressure well; in which case it may occasionally be passed over, even by an attentive and experienced practitioner. It frequently happens, towards the end of this disease, that the pain suddenly ceases, and, from the relief experienced by the patient, she indulges herself in the vain hope of perfect recovery. This calm, however, can never mislead a practitioner of any standing in his profession.

"There is more or less tumefaction of the abdomen, (even before any effusion takes place,) which in general proceeds from a tympanitic affection of the bowels. Some suppose this to be a symptom peculiar to the disease; which is quite an error, as it occurs not only in every inflammatory affection of the stomach and bowels, but in every slight tendency to such a state. Diarrhœa diminishes this tympanitic affection.—(p. 35, 36, 37.)

Some desultory remarks on what the author calls the tricks of certain members of the profession, follow this description; after which Dr. Mackintosh enters at more length into the symptomatology of the disease. We here again meet the often-repeated expressions of "I have met with this symptom in several cases,"

—"I have seen several fatal cases of puerperal fever;" which expressions, contrasted with the declaration of the author before alluded to, that, where he himself attended the lying-in patient from the first, he never had but one case of either variety of the fever in question,* have, we confess, a singular effect on our mind.

Treating of the pathology of the second variety of puerperal fever, Dr. Mackintosh endeavours to show that it is nearly the same as peritonitis occurring in the non-puerperal state, and as some forms of dysentery. To the first part of this position, (which, by the by, has been already successfully urged by we do not know how many writers on the subject,) we have no objection; but we cannot so readily admit the second part of it: neither can we for a moment entertain the author's assertion, that he has, as "he humbly conceives," satisfactorily established that the phenomena of the first, or congestive, variety of the puerperal fever bears a striking analogy to other congestive diseases. We have already proved the contrary, to the satisfaction, we doubt not, of our readers; and, as our author, in the whole extent of his treatise, has only mentioned one solitary case of this form of the disease, (which was not even attended by him, and of which the outlines only are given,) we contend that, with such a *modus probandi* in medicine, nothing can be "satisfactorily established."†

Returning now to the consideration of the second variety of the disease:—As Dr. Mackintosh has, in fifty places, alluded to his great experience in puerperal fevers, especially during the late epidemic of Edinburgh; and as he has thought proper to write a book upon it of upwards of 300 pages; our readers will, naturally enough, expect to hear that he has given an account of a great many cases of it, successful or unsuccessful, but particularly of the former description, boasting, as he does (page 261), of the great success he has experienced in the treatment of the disease under consideration. If such be their expectation, we are sorry to inform them that disappointment will follow it. The only cases given at full length by Dr. Mackintosh, in any way connected with his practice, (of course we do not allude to those he has borrowed or reprinted from almost every writer on the subject,) are *eleven* in number; and of those cases Dr. Mackintosh saw only the dissection of the

* Does not this declaration convey a tacit censure on those accoucheurs who, having had an opportunity of attending a lying-in patient from the first, have been unfortunate enough to see puerperal fever follow delivery? and on none more strongly than on his colleague Dr. Campbell, and on that gentleman's pupils.

† "And it is abundantly absurd in any one, *to draw conclusions, and publish them,* upon *the experience* derived from a *solitary case.*"—Dr. Mackintosh, p. 42.

first; the second and third are of an old date, having occurred in French women who were living with English soldiers, when the author, we presume, was serving in the army; the fourth occurred in Berbice, in the year 1810; the sixth, seventh, eighth, and eleventh, belong to Dr. Campbell's practice; while the ninth and tenth, besides two cases of fever after abortion, are peculiarly the Doctor's own.

Thus far as to the extent of our author's experience, as stated by himself. With regard to *his great success,* we must imagine that he had some good reasons for keeping back all the successful cases he may have attended; for, were it fair to judge from the result of these identical eleven cases, we should not be able to join our author in the compliments he himself pays to his good fortune. Of the eleven cases detailed,—not including the two cases of fever after abortion,—six proved fatal!

Before we leave Dr. Mackintosh's work, we will recapitulate, in a few words, our observations on it, lest our readers may deem us too captious.

I. The author's principal aim, throughout his volume, is to establish two great and striking divisions, or varieties, of puerperal fever.

A. The first variety he assimilates to what he calls *congestive diseases,* and to what Dr. Armstrong has thought proper to term "the congestive form of fever in child-bed."

*a.* Dr. Mackintosh states that he has seen several instances of this variety of the disease, in all of which he was only called in to witness its fatal conclusion. *He never once saw the beginning* of the disease. *Ergo,* his minute description of the onset, as well as insidious approaches of this new variety of puerperal fever, with "all and every one" of the premonitory symptoms, as he himself calls them, must be imaginative.

*b.* Dr. Mackintosh moreover promises to refer, in the body of the work, to the several cases of the congestive variety of puerperal fever which he has seen, and to the "numerous dissections" he has witnessed; but concludes his volume without keeping his engagement.

*c.* Dr. Mackintosh proposes a treatment for this variety of puerperal fever, thus known, thus established, thus observed, thus explained. This treatment will prove successful, provided it be adopted in the onset of the disease. Dr. Mackintosh *never saw the disease* at its onset, except in one case. *Ergo,* the success to be expected from his treatment can only be conjectural.

B. The second variety, or inflammatory puerperal fever, is contended for and described by Dr. Mackintosh, in precisely the same manner that many preceding writers had adopted long before him. His urgent recommendation of early and repeated venesection in the treatment of this variety, is also a repetition of a similar recommendation from other authors.

II. The author's *secondary* aim in his work is to inform his readers, over and over again, that he has had great experience in puerperal fever; and that his success in treating it has been considerable.

A. The first of these assertions can only be rendered valid, not by a *simple allusion* to an indefinite number of cases seen, but by the historical and distinct relation of some of them, as well as of their treatment and result.

*a*. Dr. Mackintosh has not only failed to do this, but, in the eleven cases which he has either simply alluded to or minutely described, he has included one of which he had only seen the dissection,—three which occurred several years ago, and are very imperfectly narrated,—and four which belonged to the practice of a contemporary physician, leaving only two cases peculiarly his own; although the puerperal fever had been prevailing epidemically in Edinburgh during the last two years. *Ergo*, Dr. Mackintosh's great experience in puerperal fever is yet to be proved.

B. The second of the author's assertions, namely, that which relates to his great success in the cure of puerperal fever, like the first, can only be established by the narration of at least a dozen successful cases of the disease, treated by the author himself during the late epidemic.

*a*. Dr. Mackintosh has only related two cases peculiarly his own, those of Elizabeth Mace, and of a woman (name unknown,) which occurred during the said epidemic; and the two cases in question proved fatal! *Ergo*, Dr. Mackintosh's great success is yet to be proved.

It follows naturally, from all that we have said of the author's performance, that the words of counsel which, with all due humility, we desire to whisper into his ear at parting, must be short and few.

[To be continued.]

---

Art. III.—*A Practical Treatise on the Symptoms, Causes, Discrimination, and Treatment, of some of the most important Complaints that affect the Secretion and Excretion of the Urine. The whole exhibiting a comprehensive View of the various Diseases of the Kidneys, Bladder, Prostate Gland, and Urethra. Illustrated by numerous Cases and Engravings.* By John Howship, Member of the Royal College of Surgeons in London, Societé Médicale d'Emulation of Paris, &c. &c. &c.—8vo. pp. 438. Longman and Co. London. 1823.

Although this work of Mr. Howship's is far from being the best that could have been written upon the subject,—although it contains many grammatical errors, is frequently faulty in the composition, and not always invulnerable in its doctrines, yet it is, in our opinion, by far the best and most comprehensive of any with which we are acquainted. It will be a useful library-book to the surgeon; and its method of arrangement, and the aphoristic form in which it is written, enhance its value as a book of reference, whilst, at the same time, they render its analysis a task of difficulty, if not altogether impossible.

In a short introduction, Mr. Howship very handsomely acknowledges the assistance he has derived in his researches from his friends, especially Mr. Heaviside, Dr. Hooper, &c. The former of these gentlemen, indeed, has afforded him some of the most interesting cases in the volume, which may be said to be in a great measure the record of that experienced and veteran surgeon's practice in this class of complaints.

We shall now proceed, without further preface, to notice some of the most prominent points of this work, which is divided into two principal parts: the first treating of "those diseases that affect the secretion of urine;" and the second, "the diseases that regard the excretion of urine. The subdivisions of chapters, sections, and separate paragraphs, defy enumeration. There are ninety-one cases illustrating the diseases described, and four plates. A very copious Index concludes the volume.

The first subject treated of is *suppression of urine, and its causes*, and these are discussed in the following order: suppression from congestion, from inflammation in the kidneys, from calculi in the kidneys; and the chapter concludes with cases of abscess affecting those glands. We do not find any thing that need detain us in the symptoms or treatment of the first class of these affections. The method of relief, or cure, resolves itself into bleeding, either general or topical, the warm bath, aperients, and, lastly, blisters and terebinthinate medicines. On the application of blisters, Mr. Howship remarks, that "they are said to be hazardous in renal inflammations, from the possibility of absorption;" he has, however, frequently directed them, and almost invariably with advantage. (page 11.) Complete suppression is sometimes the consequence of repelled gout; and this circumstance should be borne in mind, because it must necessarily influence the treatment; and, upon the whole, it may be said, as a general rule, that inflammations of the kidney do not require the same rigorous and free use of the lancet which is indicated in other visceral inflammations: and the rather so, as it is a disease most commonly attacking people beyond the middle period of life, and who are affected with gout or some other constitutional taint.

In speaking of *abscess of the kidney*, which, as a consequence of inflammation, follows next in order, our author distinguishes the scrofulous abscess from that which is the product of common phlegmonous inflammation. In the former, the sympathetic irritation at the neck of the bladder he considers as the leading symptom, and the only apparent source of misery to the patient. One well-marked case only of this affection is mentioned by Mr. Howship, as having occurred in his own practice; and, upon the whole, the information upon this interesting subject is scanty, and not very satisfactory; and the method of treatment is dismissed with a few general remarks.

"The kidney is, however, subject to other chronic derangements of structure; one of the principal of which is the formation of serous cavities, or cysts, within its substance, or upon its surfaces. The most common variety of this disease consists of cavities filled with a limpid fluid, in some instances few in number, occupying the external part only; in others exceedingly numerous, and dispersed throughout the substance of the gland. I have most commonly seen these appearances between the peritoneal surface and the cortical substance of the kidney; part of the transparent membranous cyst raised above the surface, and part of its fluid contents buried within the substance of the gland. These cysts appear to originate in the fine cellular texture connecting together the solid structure of the kidney. Serous fluid is deposited at certain points, and, as this fluid accumulates, the pressure from within probably operates by condensing the cellular structure, so as to form a kind of fine cysts. As these cysts progressively increase in size or number, the solid substance of the kidney is removed by absorption, till at length, where the disease has been of long standing, scarcely a vestige remains of the natural organization. The most beautiful specimen I have ever seen of this disease, is to be found in the elegant collection of Dr. Hooper. The kidney is much enlarged, and entirely converted into a closely-disposed assemblage of cysts. A section carried through the whole exposes these cavities, from the size of a currant up to that of a walnut, without any remaining trace of healthy structure.

"Another variety of serous cysts, to which the kidney is subject, constitutes the true hydatid; where the fine membranous bags containing the fluid, instead of being intimately connected with the surrounding parts, as in the former kind, are loose and detached, so that, if the serous cavity containing them happens to rupture into the pelvis of the kidney, the hydatids may escape into the ureter, and, thus finding their way down into the bladder, pass off with the urine.

"The symptoms attending these affections are always extremely obscure, and in some cases pass entirely unnoticed. In a case mentioned by Dr. Davis, a middle-aged lady, after repeated attacks of pain in the loins, had regular symptoms of stone in the left kidney; a grinding or acute pain in the part, vomiting, the urine in the paroxysm tinged with blood, and containing small shreds of coagula. During the attack, more than a dozen hydatids were passed, thin and membranous; some an inch and a half long, the thickness of a goose-quill, and filled with a fluid, which in taste and smell appeared to be urine. The paroxysm generally lasted some hours; and, when all the hydatids had come away, which happened in the successive endeavours to void the urine, the pain in the back, &c. abated, and she continued easy and well for the rest of the day. The patient perfectly recovered.

"One of the least frequent consequences of disease in the kidney, is induration or scirrhus. This state has been described as one of the occasional effects of inflammation, and M. Desault has enumerated the symptoms with which he considered the affection might be attended. Upon none of these signs, however, should I confidently rely, as enabling the practitioner to distinguish a scirrhous affection of kidney from any other chronic state of disease.

"Should the symptoms of inflammation in the kidney suddenly give way, all local pain suddenly subside, the pulse losing its firmness and becoming unsteady, there will be ground for apprehending that, overpowered and exhausted by excitement, the increased action is sinking into a state of gangrene. This occurrence, which I have never myself witnessed, appears to be invariably fatal. It is right always to bear in mind the possibility of so serious an event, as an additional motive to activity and vigilance in relieving and moderating that excess of action which can alone lead to it.

"Provided, however, the general health is good, it is remarkable how considerable a degree of irritation the kidney will sometimes be able to support. A case proving this is related in Dr. Hennen's valuable work on Military Surgery, where a large fragment of cloth, driven by a musket-ball through the body, lodged in the kidney, inducing repeated abscesses and severe peritoneal inflammation, was afterwards traced, by its symptoms, making its way down the ureter into the bladder, and thence outwards by the urethra, after nearly eight months of protracted and severe suffering, from which the patient eventually recovered."—(p. 36—39.)

Chapter 2d, on the *morbid appearances of the urine*, commences with the appearance of blood, and which, says our author, may be derived either from the kidneys, the ureters, the bladder, or the urethra. With the expection of scurvy, the presence of calculi in the kidney is, in our opinion, by far the most frequent cause of hemorrhage from those glands. In the case of scurvy, the accompanying constitutional symptoms will sufficiently point out the source of the bleeding, and suggest the method of cure.

"A discharge of blood perhaps never takes place from the ureters, unless, from the previous irritation of calculi, they have become highly vascular, and then suffered injury. In a healthy state, their supply of blood is not such as to furnish the means of notable hemorrhage; but, where the patient labours under cancerous or fungous disease of the bladder, or where an enlarged or varicose state of the vessels about the neck of the bladder exists, bleeding may at any time arise.

"Bleeding from the urethra frequently arises from the passage or lodgment of a calculus: it sometimes takes place from the use of caustic in stricture, from the forcing a passage through a stricture, or the laceration of some part of the canal. It is not an uncommon occurrence in the inflammatory stage of gonorrhœa, particularly during the violence of chordee; and is generally the first consequence of violent contusion of the parts.

"From the symptoms, it may be generally determined, with tolerable precision, from whence the bleeding is derived. Where hemorrhage proceeds from the kidneys, it is frequently preceded or attended by pain or sense of fulness in the region of the loins; although, if the blood passes by the capillary arteries, this may not always be the case. In those affections where the loins have previously suffered by external violence, there can be no difficulty. Should the preceding history show the probable existence of renal calculi, the appearance of blood will be at once referred to its proper source; and, where bleeding takes place from the ureter, in consequence of a stone passing down, attentive consideration of the symptoms will generally remove any doubt.

"Where blood flows from the vessels of the urethra, it may be generally known by the hemorrhage either coming on, or continuing, independent of the act or desire of voiding the urine; the blood is also usually pure, and unmixed with the urine, and, on standing, forms a coagulum, bearing a just proportion to the serum, as in healthy blood. Now and then, however, these appearances are not to be relied upon; for M. Desault observes, what I have myself more than once seen, that occasionally, either from a coagulum forming, or from some other cause, blood poured out into the urethra will flow backward into the bladder: it will then induce another set of symptoms, and thus be very apt to mislead the judgment.

"Blood passed with the urine, if in minute proportion, will merely impart a scarlet blush to the urine: when, however, any quantity has flowed into the bladder, unless voided immediately, it forms a coagulum, and the efforts at expulsion will then fail. In this state of things, the retention of urine is not the least common nor the least serious consequence." (p. 52—54.)

The treatment of this symptom must, of course, be conducted in reference to the affection of the particular organ; but, when blood has become effused into the bladder, it will be necessary to adopt some means for facilitating its dissolution and removal. For this purpose our author recommends, as the most safe and harmless proceeding, the injecting into the bladder, by means of a catheter, a quantity of warm water, previously rendered perfectly soft by the addition of a little alkali. The longer this can be retained the better. In addition to this means, Mr. Howship recommends breaking down the coagulum, by means of a small silver catheter. Every movement of the instrument, he goes on to say, should be conducted with the greatest caution, and the patient being desired to say when it gives pain: the same exact direction should not a second time be given to the point, especially if there is any suspicion of the bladder itself being unsound. (p. 58.) The smaller instrument withdrawn, the largest may now be introduced, and probably some urine will flow. Three cases in illustration conclude this section.

Section 2d, *on the appearance of pus in the urine.* This section will not detain us long. We are not ourselves convinced of the possibility, upon all occasions, of distinguishing purulent from other deposits in the urine. The tests laid down by our author, and the microscopic examinations, are not always available in practice. Besides, as he himself has observed, mucous surfaces can, and do, pour out discharges truly purulent, without any breach of surface; and the detection of this latter circumstance is, after all, our principal motive for wishing to arrive at a positive decision on the subject. The following paragraph, however, in our opinion, clears up the difficulty, as far as it is capable of being cleared up, and they are entirely conformable to our own opinions.

"The opportunities of examining urine in which the matter deposited is, properly speaking, purulent, are generally in cases where the fact is already rendered clear by the other symptoms; and, consequently, the determining the nature of the deposit becomes unnecessary. In one very recent instance of this kind, a copious deposit of purulent and coagulated albuminous matter in the urine occurred in a man in whom there were strong reasons for believing these appearances arose from purulent disease of kidney. No medicines appeared either to relieve or to influence the progress of his complaint; from the continuance of which, sinking into colliquative diarrhœa, he at length died. On examination, I most unexpectedly found the kidneys and ureters perfectly sound; but an abscess formed within the psoas muscle, connected more intimately by thickening and disease of the cellular membrane, to the lateral part of an otherwise healthy bladder, had discharged its contents into the cavity by a small opening, which thus passed off freely with the urine by the urethra.

"The importance, however, of not asserting or admitting the presence of ulceration in any internal part before it actually exists, is more considerable than may at first be imagined. Any consequence of irritation, any altered action upon a natural surface, it is pretty generally known, may often be corrected; but, if that natural surface is once broken up by ulceration, the probability of its restoration, or, in other words, the chance of a cure, becomes exceedingly diminished. Now, suppose a patient with irritable bladder, and the appearance of filmy albuminous substances in his urine, is told by a surgeon of high character that there is an ulcer in the bladder, and that those substances are the coatings of the ulcer, he is depressed, but not relieved. But, should two other opinions fortunately coincide in considering his complaints derived from irritation only, and capable of being removed, he feels fresh confidence, his spirits improve, his constitution recovers the power of helping itself; and this state of constitution being, in my opinion, very often of more consequence than any medical treatment, it becomes evidently important to the patient's welfare that his case should not be set before him clothed in imaginary terrors: nor, indeed, should it be placed in any less favourable point of view than known facts will warrant. There is rarely any necessity for concealing the truth; indeed, it may always be made known in proper terms, even when unfavourable: but, where the actual circumstances remain conjectural, it appears to me a serious duty to avoid putting a strained construction upon every incident or symptom; when the only effect can be to give it additional importance, by setting it in the most unfavourable light, and the only object an idle affectation of superior discernment." (p. 67—69.)

The third section, *on the appearance of albuminous matter in the urine*, commences with noticing those deposits usually the product of general disease, &c. and particularly from gout transferred: in fact, so various are the causes, that to the skill and sagacity of the practitioner must be left the task of tracing the disease to its source. External injuries in the neighbourhood of the kidneys, irritation at the neck of the bladder, and disease of the prostate gland, are some of the local causes of these depositions, more especially coming within the range of the surgeon's practice; and which are illustrated, in the work before us, by two or three very good cases.

From page 79 to 154, the subject of *gravel* and *calculus* is discussed; but we do not find any thing of novelty to arrest our attention. Mr. Howship shows himself to be intimately acquainted with the writings of Drs. Prout, Marcet, and other modern writers; but in all that relates to the various mode that have been proposed for performing the operation of lithotomy, as well as for extracting calculi from the bladder of the male and female, he is neither so full nor comprehensive as might have been expected in a work professedly surgical: perhaps he is of opinion that these mechanical contrivances, especially, are not likely to be often available in practice; or that, in certain instances, they afford only a chance of multiplying the disease they are intended to remove; and, probably, such a view of the matter, after all, is not very far from the truth. We can, however, conceive a case, in which the application of such an instrument as Sir Astley Cooper has successfully used for the removal of small calculi from the bladder, may be attended with peculiar advantages; obviating the necessity of the operation, which, at certain periods of life, and indeed under the most favourable circumstances, is perhaps the most formidable, and precarious in its result, of any within the range of surgery; but beyond this our hopes do not extend. The length of the canal in the male,—its irritability,—the impossibility of dilating it beyond a certain extent, will always afford obstacles to the general adoption of this practice; and, with regard to the plan of breaking down a large calculus in the bladder, for the purpose of extracting it, we do not hesitate to condemn this as bad practice, unless we are quite certain of getting away *all* the fragments. We are much surprised, however, that Mr. Howship has said so little relative to the extraction of calculi from the female bladder by means of dilatation, and which he has scarcely done more than allude to. He has omitted also any description of the high operation for the stone; and his history of that operation, and the different methods of performing it, are very meagre, and will not enable the surgeon to dispense with searching for further information, and perusing many works, the substance of which he might have expected to have found here.

We come now to the fifth section, treating of *irritable bladder;* but, after mentioning the general leading symptoms of this affection, and the numerous causes from whence it originates, he says, that "a degree of irritation in the bladder is an occasional consequence of external violence, or wound," (page 156;) and this leads him to a digression on wounds of the bladder; and some instances are adduced, chiefly from LARREY'S "Chirurgie Militaire," of recovery from gun-shot and other wounds of this viscus. It is well known that upon more than one occasion a musket-ball has been lodged in the bladder, and the man has perfectly recovered from the immediate consequences of the wound. The ball then produces the common symptoms of calculus, and has occasionally been extracted from the bladder by the operation of lithotomy. Here the symptoms of irritable bladder are, in fact, in no way different from those produced by a stone. Other causes of irritable bladder are next mentioned, such as cold, diseased prostate gland, diseases of the uterus, vagina, and rectum, cancer, and fungus hæmatodes; but we do not find that stricture is insisted upon, or even mentioned, as a cause of irritable bladder; and yet our experience induces us to believe that the spasmodic affection of the male urethra, the result of repeated inflammations of its mucous lining, is among the most frequent causes of this unpleasant symptom. Eleven cases of irritable bladder, from various causes, conclude this section.

We have now arrived at the second part of Mr. Howship's work, "on the Diseases that regard the Excretion of Urine;" the first chapter of which treats of *Incontinence of Urine*, and which commences thus:

"Incontinence of urine is a complaint principally occurring in early youth, although not unknown in later periods of life. Aged persons, subject to stricture or to affection of prostate gland, are occasionally distressed by want of power to retain their urine, which, notwithstanding, is in these cases a symptom of a full bladder.

"The involuntary discharge of urine during sleep has been variously accounted for, but it seems to me that a moment's consideration will clearly explain it. The general muscular coat of the cavity of the bladder may be regarded as an involuntary muscle; while, on the contrary, the circular band of muscular fibres surrounding its neck is, to a certain degree, obedient to volition. Now, we know very well that a state of repose relaxes very much the whole system of voluntary muscles, exerting little or no influence over the system of involuntary movements. Upon this principle, it is evident an involuntary flow of urine might more readily occur during sleep, than while awake. There is, however, I believe, another circumstance tending to explain how it happens. In the majority of cases, the early age of the child prevents the ascertaining whether the urine flows during a state of positive oblivion, or whether this event takes place only under some particular mental impression. The latter state, I rather believe, is mostly an invariable condition. Intimately acquainted with a young person, in early youth long subject to this habit, he mentioned to me one circumstance that, upon these occasions, had often struck him as curious: it was, that he never at any time wetted the bed, unless, when engaged in a dream, he felt the accustomed uneasiness from desire to make water, and fancy immediately supplying what was wanting in time and place, the act of voiding it became, in point of fact, as perfectly voluntary as at any other time.

"Where this disorder occurs in the adult as a simple affection, it is generally either the consequence of some paralytic affection at the neck of the bladder, or of some violent distention of the urethra.

"Incontinence of urine in the female is sometimes induced by difficult labour. In a recent case of this kind in a young woman, who, at the request of the midwife was kindly visited by Dr. Merriman, the labour was not considered such a one as should lead to ulceration or sloughing of the bladder; but, as she had scarcely any power of retention five weeks after delivery, I was requested to call upon her, examine her state, and consider whether any thing could be done for her relief. The orifice of the urethra I found irritable and red. I first passed the smooth blades of a light pair of polypus forceps (the blades of which were rather long,) just so far into the urethra as to reach the bladder; and then very gently and slowly expanded the blades, by pressing my fingers by degrees between the handles. In two or three minutes I removed the forceps, and introduced my fore-finger, perceiving in my progress a strong and tight thread which surrounded the canal at one part, the rest of the urethra relaxing very freely. This narrow ligature at first prevented the easy introduction of the finger, till it probably ruptured, as I felt no more of it, and found more freedom in then examining the cavity of the bladder with the one finger, while I followed it by another introduced per vaginam, without perceiving any trace of wound or ulceration. A few drops of blood followed the operation. Calling some days after, I was agreeably surprised on finding that, since the dilatation of the urethra, (the urine previously almost always dropping away, but never passing in a full stream,) she had now the power of retaining nearly as long as she pleased, and also of voiding in a free and large stream, 'as in health.' She soon entirely recovered.

"Incontinence of urine, it is true, does not expose the patient to such serious consequences as are induced by retention; but it nevertheless subjects him to inconveniences extremely distressing to one who is still desirous to enjoy some of the comforts of society. The clothes, always moistened and wet with urine, acquiring at length so strong a smell as to be offensive to himself, and particularly so to all around him.

"Incontinence of urine in young subjects is generally very easily removed. All that is commonly required is to stimulate, to a certain degree, the neck of the bladder; and this is most conveniently accomplished by the application of a small blister to the loins, or, if that fails, to the perineum, the blister being for some time kept open, and dressed occasionally with the ung. lyttæ. The object is to keep up a degree of irritation at the neck of the bladder during a certain period, by which the parts are roused into action; and I believe this plan, simple as it is, will generally answer: at least, I have very frequently seen it succeed, but never known it to fail.

"Where this complaint occurs in the adult, induced by fatigue of the parts, from excessive debauchery, or perhaps the consequence of a slight paralytic affection, I know of no better mode of treatment than that just mentioned. A blister will here, however, sometimes fail; and, when this is the case, the tinct. lyttæ may be given internally, so as to answer the same purpose." (p. 202—205.)

In the instance of slight paralytic affections of the bladder, which occur in conjunction with other symptoms of paralysis, in addition to the means of cure proposed by Mr. Howship, we have found, upon more than one occasion, the best effects to follow the regular use of the catheter, twice or three times in the course of the day, relieving the bladder from too great an accumulation of urine, and permitting the muscular coat gradually to recover its tone. This short chapter concludes with a case of incontinence of urine, from using large bougies for a supposed stricture; and which we conceive to be instructive, as a warning to those surgeons who advocate the use of instruments of dimensions so enormous, and which we have seen with astonishment,—we had almost said, with horror.

"Oct. 20, 1818, I was consulted by a gentleman for stricture. He stated that, two years since, he was in town, under a surgeon of eminence in London, who told him his complaint was stricture, and passed in succession different-sized metallic bougies, for several weeks; and then directed him to take a set with him down to Scotland, and pass them occasionally for himself. He produced the instruments, and the largest size (full half an inch in diameter) astonished me; though he said he could pass it, and proposed my seeing him do so. This, however, I objected to, saying I believed what he said, that it would in general find an obstruction before it had gone far; and well it might, while the urethra had any feeling; that the only symptom of which he complained, a want of power of retention, would hardly subside while he used any instrument of that size,—it being, in my judgment, not only unreasonable, but ridiculous, to think of passing such a bougie. In looking over his case of instruments, I selected the smallest of twelve, passed it as a full size into the bladder, without the least hindrance; and stated to him it was very clear there was no stricture at present, but that he could not go a more ready way to work, to produce either that or some other mischief in the canal, than by forcing in an instrument so much beyond the natural power of the urethra to receive, as that he had just shown me. To this he replied, by admitting that the introduction of the largest size had frequently brought on irritation about the prostate gland.

"I advised that he should do nothing; considering it most probable that, by discontinuing the use of instruments, the bladder would by degrees recover perfectly its power of retention."

The second chapter, on *retention of urine*, first details some of the more rare forms of this disease,—such as retention in the ureters from calculi or hydatids; and then proceeds to consider the case of retention in the bladder, and its causes, and which he reduces to three general heads: "First, those affections in which the coats of the bladder are deprived of their contractile force, from age, excess, the abuse of diuretics, affection of the brain or spinal marrow, over-distention, inflammation or spasm of the bladder, &c. Second, affections from causes within the cavity of the bladder, fungous tumor, coagulum of blood, extremely tenacious mucous, or albuminous matter effused from its inner membrane, &c. Third, affections, the consequence of displacement either of the bladder or other viscera, producing pressure on the urethra, or tumors, which in their development produce the same effect." (p. 221.) Each of these forms of retention are exemplified by one or more cases, but which the limits of a review do not permit us to extract, though some, especially of the more rare forms of disease, are interesting and instructive. In the midst of these cases of retention, we find, from page 259 to 276, a section devoted to the consideration of gonorrhœa, in which Mr. Howship advocates the old-fashioned practice of dilution, antiphlogistics, &c. Not one word is said relative to the power of cubebs, nor the propriety of an early use of astringent injections in the first outset of the disease, and before the inflammatory symptoms have become established. In fact, Mr. Howship's plan of treatment is adapted, and well adapted, to the most aggravated form of this affection in a very irritable constitution; but it must be admitted that the majority of those who contract gonorrhœa would never submit to so dilatory a plan of cure; neither is there commonly any necessity to subject them to it. What would a young gentleman say, for instance, to his surgeon, who should tell him "that in the course of a *few weeks* the inflammatory symptoms of his gonorrhœa would subside, *the only remaining circumstance* being a discharge from the urethra?" We are afraid that he would be apt to get out of humour with this *circumstance*, and his surgeon also.

Section the 17th treats of *retention produced by an enlarged condition of the prostate gland.* On this subject Mr. Howship's opinion has undergone a change since his first publication on these diseases, and he now adopts the arrangement of Dessault, considering this diseased condition of the gland to arise either from inflammation, abscess, calculi within its substance, a varicose state of its vessels, or a schirrous disease of its substance. (p. 292.) Each of these forms of disease is treated of, generally, in a very judicious, but in a somewhat concise, manner. The schirrous enlargement of the gland is generally the disease of old age, but we have had occasion often to suspect the existence of some affection of this gland in much younger subjects, especially where gonorrhœas have been frequently contracted, and which complaint puts on very much the symptoms of spasmodic stricture. Of course, examination *per anum* is the only sure method of ascertaining the existence of this affection. We subjoin a case, which gives a good history of the common form of this disease.

"J. L., a man aged seventy-three, observed a degree of frequency in passing his water, in May 1816. He soon afterward complained of being occasionally unable to pass it at all, even when he had left his bed for that purpose, until he had walked a little about the room.

"April 23, 1817.—He could pass no urine, though distressed with frequent desire and straining. On the 25th, he desired a medical gentleman to see him, who ordered the warm bath. He had still urgent desire, but passed nothing. On the 26th, I was requested to visit him, and, introducing a large catheter with perfect ease, drew off more than two quarts of water. The prostate gland, examined by the rectum, was found considerably enlarged. From this time I continued to draw off the urine regularly till the 22d of May, when he recovered the power of expulsion. The water generally exhaled a strong ammoniacal odour, was of brownish colour, without mucus, but sometimes depositing dark-red particles of gravel.

"August 17.—He called, and said he had some returning difficulty in getting rid of his water, generally worse towards evening, and better on the approach of morning. Some leeches were directed to the perineum.

"Oct. 20.—He sent in great distress, not having passed any water for two days. I drew off two pints, and the retention left him.

"Jan. 28, 1818.—He sent to beg me to see him as soon as possible, having passed no water for the last thirty-six hours. I now drew off three and a half pints; after which the bladder recovered its power. It appeared that the last attack had been the consequence of some degree of intemperance.

"Jan. 1, 1819.—For several days the retention had returned. I again examined by the rectum, and found the prostate as large as the half of a middling-sized apple, so that the finger could scarcely compass its convexity, or distinctly reach the bladder above it.

"Sept. 3.—From severe cold, the retention returned, and had existed twenty-four hours when I visited him. The silver catheter found some obstruction from spasm at the bulb of the urethra, but, with some little pressure, passed on to the prostate; and here a finger, behind the scrotum, easily raised the point over the projecting part of the gland, and it readily slipped into the bladder. The water drawn off for two days, he again recovered the power of voiding it by the natural efforts.

"Jan. 26, 1820.—Had a return of retention, which resisted the introduction of the catheter with more than usual obstinacy, the prostate gland feeling of a cartilaginous firmness. By keeping the instrument in the bladder for a few days, he recovered. In June and September following, I was again obliged to relieve his full bladder, after which the power of expulsion returned.

"Nov. 1822.—He continued in good health, although occasionally obliged, for a day or two, to have recourse to the catheter."

The two following sections, on *retention from spasmodic stricture*, and on *retention from permanent stricture*, occupy nearly one hundred pages, including the details of upwards of twenty cases. There is nothing under these heads of disease that we feel it necessary to transcribe; and the length to which this article has already extended warns us to hasten to a conclusion.

The last section, on the *puncture of the bladder*, is short. Mr. Howship prefers performing this operation through the rectum; although he admits that, "where retention is consequent to disease, with much enlargement of the prostate gland, the puncture is more conveniently made above the pubes." (p. 413.) We shall conclude by giving our author's description of performing this operation through the rectum.

"In puncturing the bladder from the rectum, the trocar need not be much larger than that employed for hydrocele, although it must be twice the length, and should have a gentle even curvature. The form of the instrument, with the exception of its curve, should be that of the common trocar, with a triangular point, for the reasons already given.

"As to the position of the patient, it is not exceedingly material; the operation may be conveniently enough performed as he lies in bed. The fore-finger, well oiled, is to be first passed up into the rectum, and the degree of fulness of the bladder, as well as the most convenient point above the prostate gland, ascertained. When these circumstances are satisfactorily made out, the trocar pushed nearly, but not quite, through the canula, is to be gently introduced through the sphincter of the anus, and passed up until the extremity of the canula, corresponding in situation with the point of the finger already in the rectum, is felt to be against the part where the puncture is to take place. The canula being in the least degree retracted, while the stilet is pressed forward, places the instrument at once in a fit state for effecting the puncture; keeping in view the line of direction tending to the centre of the distended bladder, the trocar is now to be steadily passed forward through the coats of the intestine and bladder; when, the stilet being carefully withdrawn, the canula must be retained, and the urine allowed to flow off.

"As the accidental slipping of the canula out of the orifice in the bladder has been productive of inconvenience, from the premature healing of the wound, and the consequent necessity for repeating the puncture, it is desirable to have a little plate attached to the external part of the canula, perforated with holes, so as to admit of its being secured to a bandage, passing round the waist and between the thighs. This precaution, however, generally becomes unnecessary after the first week or two, as the opening usually very soon loses its disposition to heal, until the restoration of the natural passage for the urine renders it useless." (p. 413—414.)

---

ART. V.—*A Series of Elementary Lectures on the Veterinary Art.* By Veterinary-Surgeon PERCIVALL, of the Royal Regiment of Artillery.—8vo. pp. 377. Longman and Co. London, 1823.

THERE is an interest attached to the anatomy and physiology of the horse, proportionate to the value of the animal to man, independently of the study of its structure and vital economy as an object of comparative anatomy. Great encouragement is therefore given to the improvement of the veterinary art, by opening all the resources of medical science to its professors and their pupils. Consequently, the education of students in farriery is placed upon the same footing as that of the students of our anatomical schools. Thus, whilst the art acquires dignity, the rank and emoluments of the veterinary surgeon partake more of the advantages enjoyed by practitioners in human medicine than formerly.

We rejoice in any indications of a disposition to rescue the treatment of our domestic animals from the barbarism with which it has been so long enveloped, and to substitute practical deductions from rational principles for remedies sanctioned by the experience of grooms and jockies.

Mr. PERCIVALL is one of the modern school in the veterinary profession, and he has presented to its notice a series of elementary Lectures upon the Anatomy, Physiology, and Pathology of the Horse, according to the principles of human medicine, as applicable to farriery; and his work is interspersed with observations derived from the sources of his own experience. We are not aware that the author has been anticipated in any systematic treatise of this kind, although there are many before the public upon detached subjects of farriery and the structure of domestic animals, especially of those organs for the employment of which they are chiefly valued. The author thus explains his objects, and the resources from which he has drawn his materials:

"With all that was known prior to the institution of the College,—with that vast accession of convertible science for which we are indebted to human medicine,—and with what has since been added by later writers, we purpose to compile a regular course of Lectures on the Veterinary Art; parts of which we shall submit from time to time, until the whole be completed, to the perusal of those desirous to acquire an elementary knowledge of it. For students we principally design them: should they, however, be deemed worthy the attention of others, we shall feel amply repaid for our time and application. Our physiology we adapt to the popular doctrines of the day. To compose our pathology, we avail ourselves of the labours of those few who have written from their own observations; and we have copious notes of our own to refer to, as well as a large assemblage of records, collected by ourselves and others in the course of a very extensive practice, of nearly thirty years' duration, in the service of the Royal Board of Ordnance. * * Our aim is to lay down *principles fundamental to the attainment of a knowledge of the veterinary art.*"

This promises fairly; but we see no reason why the author should write in the plural number, considering that he is speaking for himself individually, and not for a body.

With regard to the general arrangement of the system, although it must be allowed to be perfectly arbitrary, yet we should prefer commencing with the functions of the nervous and muscular tissues; and we do not see the assumed advantages of affording the *blood* precedence, before the student is made acquainted with the two great causes upon which its circulation depends.

On the subject of *animal heat*, the author states that it is regulated by the temperature of the surrounding medium. This does not accord with the vital principle, which resists those changes of temperature that inanimate matter is subject to. Mr. Hunter discovered that the belly of a carp, in a frozen pond, raised the thermometer far above the surrounding temperature. A similar phenomenon exists in the interior of frost-bound trees. A hen's egg is known to retain a certain degree of heat above that of the air. As a specimen of the author's style, we insert the following passage on the *diseases of the blood*:

"All diseases were formerly supposed to originate in a morbid condition of the fluids of the body; from which notion, such incorrect ideas were formed of their nature as led to a mode of practice, either indirectly injurious from its inertness or want of efficacy, or positively pernicious from its co-operation with the morbific agents themselves. The ancients supposed that there was some peccant or offending matter floating in the fluids, or, as they called them, humors of the body; and that nature, requiring the expulsion of this imaginary acrimonious matter, instituted a diseased action in the system for that purpose; which was occasionally to be promoted, but never suppressed, by the practitioner. To this doctrine, which is called the humoral pathology, the veterinary art has long been subservient; and, though it has been of late years in a great measure rid of such obscure tenets, still are the *humours afloat* among our crafty vulcanians of modern times, whose practice as often tends to exasperate as to relieve the sufferings of their unfortunate patients, however well adapted it may be to impose on the credulity of their unsuspecting employers.

"The explosion of the humoral pathology has been followed by a doctrine, that, although the blood in disease exhibited morbid signs, it was never diseased in itself; or, in other words, that it did not contain any morbific ingredient. The principal arguments advanced in support of this opinion are two: viz.—first, that, if it were diseased, as it flows to every part, the morbid matter it contains ought to create general, and not local, irritation; and secondly, that, if it were so in contagious diseases, we ought, by means of inoculation with it, to produce the same effects as arise from the introduction of the contagious matter itself. In attacking the first of these positions, we would ask, if there be any disease which invades alike all parts of the body? and why, on the contrary, so many seem to have a predilection for particular parts? In the human subject, for instance, small-pox and measles confine themselves to the skin; scrofula affects the absorbent system; and the venereal disease, either the organs of generation, the throat, the skin, or the bones. Again, in the horse, farcy is an affection of the superficial, not of the deep-seated, absorbents; and glanders, one confined to the membranes, &c. of the nose; and yet all these are, with the exception of scrofula, contagious diseases, in most of which, if not in all, probably, the circulating mass is contaminated. The second of these arguments admits of a more ready reply. No one ever imagined that the morbific matter contained in the blood existed in so concentrated a form as to produce its effects by inoculation with a single drop of that fluid; no more than that the venereal virus, diluted with fifty or a hundred times its weight of healthy pus, would convey the disease by inoculation with a fiftieth or hundredth part of such a mixture. In further illustration of this, we can, and do medically, not only without any baneful effect, but with manifest advantage, take minute quantities of substances of so poisonous a nature, that the old practitioners were altogether deterred from the use of them. This subject, however, has of late been put without the pale of disputation by actual experiment. Some years ago, at the Veterinary College, the blood of a horse affected with glanders (which is a contagious disease, and one at all times readily communicable by inoculation,) was transfused into the veins of a healthy ass, previously prepared by copious blood-letting: in the usual space of time, the animal exhibited every symptom characteristic of glanders, of which it shortly died. Considering the antiquity of the practice of transfusing blood from the vessels of one animal into those of another, it is somewhat remarkable that this fact has never been before demonstrated by experiment; for it is now nearly two centuries ago since this operation was first attempted on dogs, and subsequently on men, with expectations of the most glittering description: like those who were so solicitous about transplantation of teeth, however, the individuals on whom it was practised were occasionally seized with diseases of a malignant and dangerous tendency.

"The blood not unfrequently, in the human subject, exhibits what are considered as morbid appearances: whether this be the effect of what Mr. Hunter calls 'contiguous sympathy—a real increase of animal life," or whether it arise from some variation in the relative proportions of its constituent parts, or be produced by some morbific matter contained in it, we are not about to inquire. All we wish to impress here is, that what is regarded as indicative of increased, if not diseased, action in the vascular system of the human subject, is perfectly natural to that of the horse in his domesticated state: if, therefore, you examine the blood of a horse in apparently good health, you expect to find it sizy. It has been, with great truth, remarked by some of our best practitioners in human medicine, that an inflammatory diathesis is by no means essential to the production of buffy blood; an opinion confirmed by the appearances of horses' blood in health. We do not believe that this fact is universally known: we have searched the works of veterinary authors on the subject, with but little advantage; for they have either transcribed their accounts, which are for the most part erroneous, one from another, or borrowed them from human anatomy." (p. 24—27.)

The old question of the life of the blood itself is disposed of according to Mr. Hunter's notions. Indeed, all which has been said against these seems to us to amount to a mere *quibble*. The blood may be said to be alive, because it circulates in living vessels, carrying with its streams the means of growth, of reproduction, and the support of life; and, when it ceases to be circulated, its characteristic qualities are destroyed.

Respecting *vascular action*, the author believes arteries to be irritable. Near to the heart, he thinks, the blood is propelled by that organ, but that further off its influence is lost; and that here the arteries themselves urge on the blood, by the muscular contractility opposing the distention of the elastic coat. The phenomena of the circulation in general favour this idea.

In the lecture on the *brain* and *nervous system*, the nerves are described as of a "*soft substance;*" a term which scarcely conveys an accurate notion of their real feel, especially in the horse, although some nerves may be firmer than others.

The author speaks of the *spinal cord* as possessing sensation, like the brain. From the great mass of contradictory evidence before the public, it is not surprising that one, endeavouring to cull the ore from the dross, should feel himself embarrassed as to the proper arrangement of the functions of the nervous system. We must say, after sifting this matter well, we are disposed to consider the brain as *exclusively* sensible; and we think M. Flourens has satisfactorily shown that the seat of sensation is limited to a *particular portion of the brain;* the spinal cord, like the nerves, being merely the conveying medium of impressions.

The author notices the distinct properties of nerves generally, but we think he is not sufficiently explicit upon the interesting subject of the appropriation of some nerves to *sensation*, and of others to *motion only*, which has of late been much elucidated by experiments.

Proofs of the regeneration of nerves are cited, in cases of previous division. Simply cutting a nerve in two often proves fruitless some time afterwards, so that, in the operation of neurotomy on the horse, a piece of the nerve is now cut out. We have still doubts of the perfect restoration of nervous matter and sensation, after division has taken place along the course of the original nerve. It may happen, in time, that the re-union is capable of conveying *slight* impressions, as we ourselves have witnessed. We also, from our own experience, differ from the author as to the contractile powers of nerves. The divided ends have always appeared to us to *overlap* each other, rather than retract.

With reference to the passage of nerves, the author seems to think that they may either be traced *from* or *to* the brain. We certainly admit the latter; and the former has been the commonly-received mode of considering the nerves. It has long appeared to us that we have been wrong in describing the nerves as *originating* in the brain; and that it tends to elucidate many of the phenomena of nervous action, to trace them *from* the different organs of the body, as their proper source and origins, and consider them as *terminating in the brain.* Instances of congenital hydrocephalus are recorded, in which the nerves were perfectly formed, and their extremities found within the cavity of the head, in peduncles bathed in the surrounding fluid. In such cases it is difficult to imagine them generated in the head.

A whole lecture is devoted to the operation of *neurotomy*, in favour of which Mr. Sewell has warmly combated; and we must say that the objections to the operation have savoured more of prejudice than of a scientific disposition. We ourselves can bear testimony to the simplicity, the efficacy, and the humanity, of the operation; and, from inquiry, we entertain no doubts of the perfect safety with which horses may be employed after the operation; although, perhaps, we should prefer devoting them to such duties as did not require speed, and to draft rather than to saddle. We do not hesitate to say, it is a discovery of great value to the government, to the farmer, and to all employing draft horses; whilst it enables the sportsman to retain a favourite hunter, which humanity would otherwise urge him to destroy. Of the Assistant-Professor's claims to the discovery, advocated by the author with a "special retainer" zeal, we cannot be competent judges. He has assuredly brought the operation into vogue, and improved it, and the practical results of his great experience are indisputable proofs of its efficacy.

Amongst other advantages, it is somewhat curious that a mare of great value would not take the horse whilst suffering under disease of the foot, but subsequently to the operation she became an excellent brood-mare. In another case, a stallion refused all the mares presented to him. He laboured under disease of the foot, and, when the operation of dividing the pastern nerve was performed, he then became a useful stallion. An instance also occurred in which tetanus was obviated by this operation.

We quote the author's description of the method of performing this operation:

"The operation is by no means difficult to him acquainted with the anatomy of the part. The horse being cast, and properly secured, and the affected limb extended and placed in a convenient position for the operator, an incision, about one and a half inches long, is to be made upon the side of the large pastern bone, opposite to, and in the direction of, the large pastern nerve; then, having cut through the cellular substance, so as to lay bare the trunk of this nerve, (anteriorly to which, and in contact with it, lies the artery, which must be carefully avoided, raise it with a tenaculum, and divide it by the introduction of a probe-pointed bistoury, as high up as the external wound will admit: lastly, clean it from surrounding adhesions, and detach it from the continuation of the trunk below, so as to excise a portion of about three-fourths of an inch in length. The operation requires to be performed on each side, there being two separate trunks below the fetlock; and of course on both legs, should both be affected. To close the wound, we rather employ adhesive plaster than suture, with straps of which the pastern may be encircled, if the hair be closely shorn off: where suture is preferred, the interrupted is the best, and about three or four stitches are sufficient. The legs should be bandaged* after the operation, and the horse not allowed to lie down during the first night, or be moved for the three or four following days, lest the parts be disturbed, and union by adhesion defeated.

"Though the operation admit of very extensive and diversified ap-

* "We commonly dip the bandages, previously to applying them, in the saturnine wash. It is also a good practice to give a dose of physic, and keep the horse, during his confinement, on bran, instead of corn. We may observe that, in many cases, the bandages only are sufficient."

plication, its subjects have been from the first represented to be, what all subsequent experience has shown they ought to be, *horses incurably lame;* by which we mean, *cases that will not admit of relief by any other means.*" (175—176.)

The author has devoted some attention to the gait with which horses are sometimes affected, called the "*stringhalt.*" He promises some experiments to ascertain the cause of this disagreeable motion; but it is his present opinion that it is referrible to the spine, horses being very liable to injuries of the loins, often escaping notice.

Upon the subject of *secretion*, the author refers this function to the influence of the nervous system, but in what manner or degree he does not pretend to determine. Instances are adduced of the hoof growing after all sensibility is lost from division of the pastern nerve. On the other hand, we know that the secretions of the stomach fail after division of its nerves, and digestion does not go on if their agency be removed. It is also well known that secretion is under the influence of the emotions and passions of the mind.

Our limits prevent a fuller investigation of the various interesting subjects discussed in the volume before us. Our notices have been chiefly directed to the physiological part; but, although we are less competent judges of farriery practice, we have nevertheless been led, in perusing the details of this, to consider them as deduced from sound and rational principles. We look forward with pleasure to the completion of this work. It will, no doubt, prove a useful book in the hands of those for whom it is chiefly compiled; and we recommend it to gentlemen who are not disposed to trust the care of their horses' constitutions to farriers and grooms, and who are desirous of acquiring a knowledge of the principles which form the basis of the veterinary treatment of our domestic animals.

### DIVISION II.

### FOREIGN.

ART. VI.—*Leçons sur les Epidémies et l'Hygiene publique, faites a la Faculté de Medecine de Strasbourg.* Par FR. EMM. FODERÉ, Professeur a cette Faculté.—pp. 523. A Paris, chez Levrault, 1822.

[Concluded from page 168.]

WE now resume our consideration of M. FODERÉ's work, commencing with the study of diseases during their formation, and the methods of cure.

A long chapter on *life in a state of health* introduces this subject. " Life (says our author,) cannot be substantially defined, because we only know it through its phenomena; but the idea we form of it is, that of a power always active, and directly opposed to the contrary state of absolute inertion, in which we suppose inorganic bodies to be, where we recognise only the juxtaposition of molicules and atoms in a regular or confused order. This state of inertion is death."

After indulging a few fancies relative to the analogy between the effects of life and heat, (which, however, he is *très éloigné* from thinking to be identical principles,) our author, at p. 271, sums up his creed as follows: "Since the word must be pronounced, I do not fear to avow, as I have done in former works, and as I shall continue to do in future, that I am convinced of the existence of a particular principle pervading all being: a *vital principle*, in fact, independent of the existence of the organs themselves; or, rather, that by which the organs exist, and without which they would cease to exist; a *material principle*, subject to general laws," &c.

This opening naturally leads to some metaphysical speculations, which however he promises to use sparingly, and apply with sobriety; for, as he truly observes, his business is chiefly with the phenomena of life, and not with its principle, which he acknowledges not to understand. In explanation of these phenomena, he instances the wonderful processes of the growth of a grain and the fecundation of the egg; and the effects of sensibility, excitability, and mobility, as displayed in the performance of all the animal functions,—such as nutrition and sanguification, circulation, respiration, &c. &c. All these subjects are separately treated of, as might be expected in an elementary work; but there does not appear to us any novelty of doctrine, or that which calls for particular notice.

The second chapter, on *life in a state of disease*, and on the surest means of recognizing this state, will afford us more room for quotation and remark.

M. Foderé commences by lamenting the fallacy of *the facts* that are perpetually brought forward to favour particular views of disease; and yet, to observation at the bedside of the patient, to post-mortem examinations, to dissections, and experiments on living animals, after all, we must trust for our knowledge as physicians. Our author is much disposed to place the simple observation of disease far above the other sources of our knowledge; and certainly, he observes, the treatment of inflammation was perfectly understood long before there was any question as to pathological anatomy, or of the doctrines so contested in the present day; and no physician, even the least instructed, thought of treating a peripneumony by stimulating and hot medicines. He again instances the treatment of syphilis, as arising from mere observation, without our knowing either the nature of the poison, or the tissues it especially attacks. In short, he says, and we must acknowledge that he truly says, we know not the nature of small-pox, or of the vaccine virus; and we are only able to say by experience, that such and such results will follow under certain circumstances. We think our author rather depreciates too much the information derived from post-mortem examinations: they certainly have not thrown, and perhaps are not likely to throw, much light upon the causes of tetanus, hydrophobia, and other nervous affections; but, however little the information derived from one individual case may appear, it is only by the constant recurrence of particular appearances, combined with a certain train of symptoms during life, that we are enabled to form a rational view of the nature of a disease, and of the appropriate mode of treatment. We need only instance the better treatment of fever, of certain diseases of the brain, of affections of the liver and of the heart, to prove this position: nay, to this source must be ascribed the improved practice of Broussais and his school, (for, notwithstanding he rides his hobby too hard, we think it must be admitted that his views of fever generally are more sound, and his practice more rational, than that of his predecessors,) of Rasori in Italy, and Marcus in Germany; for, though it be true that the appearances quoted by them had been formerly remarked by Morgagni, yet this is an additional proof of the value of attentive observation, since Morgagni and his school were prevented, by the doctrines of their day, from turning their knowledge to much practical advantage.

Upon the whole, as a public teacher, we do not think M. Foderé shows much judgment in speaking of pathological anatomy in the terms of depreciation which this chapter abounds in. In the treatment of chronic maladies, he declares it to have not given us even a ray of light; and he instances the treatise of M. Lallemand and Rostan upon the Softening of the Brain to prove his position. These authors, he continues, give us only a catalogue of symptoms arising from this condition of the brain, which are equally the consequences of a general affection of that organ, totally different from a softening of its substance, or even from a disease of some other viscus. This is true; but it is very possible that close observation of the patient during life, and attentive examination after death, will, at no distant period, afford some diagnostic sign at present overlooked, and which will probably designate the true nature of the affection, and thus add another triumph to pathology. The following remarks, however, are judicious, and the student should always bear them in his mind, if he wishes really to extend the boundaries of his profession: "You must observe with impartiality; you must not bring to the anatomical examination preconceived notions, which cause you to see in the dead body every thing *you wish to see,* and which makes that appear red to you which is pale to another,—which causes you to see ulceration where others can scarcely discern a spot," &c. &c. (p. 311.)

It is necessary to bear in mind, as M. CRUVEILHEIR has observed, that the greatest number of diseases are the effects of disordered vitality, before they become organic, and it is not until they have reached that condition that they come within the sphere of pathologic anatomy.

On the subject of experiments made on living animals, M. Foderé descants with considerable freedom: he doubts the advantages derived from these experiments,—he questions their analogical application to the human subject,—and he rejects their inferences in many cases.

By observation, our author does not simply mean the contemplation of the symptoms of a disease, but also the study of those changes produced in the living solids by their manner of receiving impressions, and re-acting upon those impressions; the knowledge of the uninterrupted action of the vital energies in a state of disease, in order to arrive at a termination successful or unsuccessful: in fine, the knowledge of those sympathetic phenomena of the habits and periods of a state of disease, so necessary for the choice and proper application of remedies.

Speaking of the contra-stimulant doctrine of the modern Italian school, our author observes that RASORI, TOMMASINI, and others, having observed, in certain fevers, that emetics, kermes, and other preparations, have not produced stimulating effects, came to the conclusion that they were direct *counter-stimulants.* Thus, Rasori has by degrees exhibited tartar emetic to the enormous amount of two drachms in the day, without producing vomiting,* excepting in the first instance. When the patient gets better, the emetic property again comes into operation, and then the remedy is left off. M. PESCHIER, of Geneva, has imitated this example: he declares bleeding to be useless, and that he cures all fluxions of the chest with tartar emetic only, which he gives in doses of fourteen grains in the day, without producing vomiting. These wanderings of genius, however, may, and do, lead to good. The excess of the doctrine of irritation has induced us to look with attention to those cases where the appearances of weakness are only illusory; and the doctrines of the contra-stimulants teach us how different are the modifications of sensibility in a state of health or of disease. It has long been considered as a favourable prognostic when

* In the last Number of the *Edinburgh Journal,* Dr. DUFFIN informs us that he swallowed from twenty to twenty-five grains of tartar emetic by mistake, and which does not appear to have produced any remarkable symptom.

medicines administered produce their ordinary effect, and the contrary when they either do not act at all, or operate in a manner the reverse of what is usual. In mentioning some examples of this kind, we are surprised to find M. Foderé declaring that mercury is trust-worthy in cases of hydrophobia, and asks, very gravely, if that effect is to be attributed to a specific property, or to its producing salivation? That it is used in certain fevers, and in hepatic affections, in enormous doses, and with great success, by the English, he admits; and he concludes this chapter by mentioning the case of a female who had *engorgemens tres-sensibles du foie* and jaundice, and to whom he gave some pills containing weak doses of calomel. To his great astonishment, on the third day both the jaundice and the *engorgemens* were gone; but the patient's mouth was inflamed, and she was salivated for fifteen days. She had taken only six grains of calomel.

The third chapter treats of *vital action in a state of disease, and of the judgment of, and crisis of, disease.* M. Foderé begins this chapter by remarking that diseases are capable of division into four great classes,—that is, very acute, acute, consecutive chronic, and primitive chronic disease: epidemics only are found to belong to the two first classes. There are, says our author, some causes of destruction that operate with such rapidity, that the vital power is extinguished, and no re-action takes place; and he instances the plague, yellow fever, and some other malignant fevers of Europe; and we may here add that we have frequently witnessed this effect in the fever of Walcheren; and we have seen more than one example of a robust man dying in the first seizure of this disease, never, in fact, recovering from the first impression of the cold fit. In common cases, however, there is an evident struggle of nature, announced by an increased general sensibility, and which appears in the form of anxiety, inquietude, or irascibility, according to the form of the disagreeable sensation we experience. Pursuing this train of thought, M. Foderé continues thus: "If life in the enjoyment of health is a power always active, it is no less so in disease; the difference is only, that its actions are irregular. Where violent irritation exists, the juices secreted or exhaled become the materials of new organs. It is not without astonishment that, after an acute inflammation, which has lasted but a few days, we see in the dead body a profusion of membranous productions; new bodies organized, the vessels of which may be injected; tunics thickened; viscera confounded into one mass by adhesions; and, in certain cases, a complete change in the nature of the tissues." (p. 328.)

In diseases of longer duration, our author has seen new products no less extraordinary. He mentions one instance of a lady who became covered with warts, which were almost seen to grow at the accession of each paroxysm of an intermittent, which lasted five months, and which dissipated as rapidly on the cure of this fever by the bark. From experience, he recommends that the appearances of the nails, the teeth, and even the hair, will afford information to the physician ; and he instances one of his own children, whose hair is curly and very fair, which becomes straight and changes colour, when the child is indisposed. Of this activity of the vital power M. Foderé gives many examples, and which will readily occur to the instructed reader, in the cases of the growth of fungous tumors, the regeneration of bone, &c.

Inflammation being the instrument of which nature avails herself in separating the dead from the living parts, expelling foreign bodies, and in the re-union of divided parts, the instance which he adduces of the expulsion of a portion of the intestinal canal, with a complete re-establishment of the digestive functions, though extraordinary, is not unique; and is accounted for by our author in the only way by which it is capable of being understood,—namely, by the invagination of a portion of the gut, and which, being destroyed by gangrene, are separated from the rest of the canal, and expelled by the anus, without the continuity of the passage being interrupted.

We now come to the consideration of *crises*, of which our author undertakes the defence ; but here we cannot help thinking that he is fighting with a shadow. No one doubts that certain diseases (the exanthemata, for example,) run a certain course. We may admit that, in many instances of erysipelas, the general symptoms give way as soon as the local malady is formed; which M. Foderé may, if he pleases, call *an expulsion of morbific matter:* but what, excepting the facts, do we learn from these examples? Are we not to prescribe for a disease according to its symptoms ? or are we to wait until nature, or the critical day, expels the disease ?

The study and knowledge of critical evacuations resolves itself into an acquaintance with certain phenomena of the secretions and excretions of the patient, and which, as our author observes, seldom take place in one organ only: for example,— the urine deposits a sediment, the skin becomes moist, the tongue clears up, and the bowels become open, nearly at the same time.

We shall conclude our extracts from this chapter by giving some of the critical signs annexed to the following evacuations: —Nasal hemorrhage is often preceded by throbbing of the arteries of the temple, redness and burning of the face and eyes, tinnitus aurium, flashes of fire passing before the eye, irritation at the nostrils, &c. Sweats are indicated by a preceding suppression, both of urine and fæcal evacuations; the upper part of the body more red and hot than ordinary; during sleep, the patient dreams *sometimes* that he is in a bath, as, previous to the occurrence of hemorrhage, he occasionally dreams of fires, of red colours, &c. Critical evacuations, by stool or vomiting, may be foreseen or expected. If the patient is not subject to bleedings from the nose, or sweats with difficulty, wamblings in the abdomen, weight and dull pain about the loins, and afterwards in the lower part of the belly, precede the alvine discharge. If the head be heavy, if there be a gnawing pain at the stomach, frequent nausea, and bitterness of the mouth, a hard and contracted pulse, inclination to spit, and a trembling of the lower lip, vomiting may be expected; and this crisis is usually accompanied with an abundant flow of urine. When the parotids become critically affected, a diminution of the general symptoms, followed by an unusual pain about the lower jaw, and a hard tumor of the part, sufficiently denote this termination.

On the subject of *prognostic*, M. Foderé speaks judiciously, but what he says is not novel. Generally we would caution the young practitioner to think maturely and soberly before he ventures a positive prognostic, and particularly when the complaint, however slight, presents any irregularities in its appearance or progress. We shall give an example of our meaning, by relating the outline of a case that occurred lately to a highly respectable practitioner, of whose judgment and professional knowledge we entertain the highest opinion. This gentleman was sent for, in the evening of one of the months of last spring, to see a married lady, who had passed the morning in her usual avocations and health, and who had dined afterwards heartily. Soon after dinner she was seized with a pain in the epigastric region, gradually increasing in intensity, and which induced her to send for her medical adviser. On examining the patient, it did not appear that the pulse was at all affected; there was neither heat of skin nor thirst; no irregularity in any of the animal functions, but an acute pain in the epigastrium, not increased by breathing or pressure. The patient was bled, however, and some medicine administered. On the following morning, when the medical man repeated his visit, to his astonishment the patient was no more. The account he received was, that, a short time after he had quitted the house the preceding evening, the patient, upon being questioned as to the pain at the stomach, declared it to be better, but expressed an unwillingness to be disturbed, and wished to lie down on her bed; soon afterwards she became faint, her countenance altered suddenly, and she soon ceased to breathe. Dissection showed the cause of this sudden and melancholy event: a blood-vessel in the stomach had given way, and the cavity was filled with upwards of two quarts of coagulated blood. Surely this may be considered as a very instructive case to those who are habitually rash or sanguine in the prognosis of maladies.

M. Foderé's fourth chapter is devoted to a consideration of the reciprocal relation of the *solids* and *fluids*, and on the influence of *habits* and *periods* in a state of disease: this consists only in knowing how to distinguish, among the phenomena of disease, the primary from the secondary, which M. Foderé designates by the title of pathologic sympathy, and of which the affections of the stomach and head offer the strongest, and perhaps the most frequent, example. The countenance, says our author, is more altered by diseases of the bowels than of any other part of the body; and he applauds the maxim of BAGLIVI, who says that if, in chronic diseases, the face preserves its natural expression, there is no reason to apprehend organic affections of the viscera.

On what M. Foderé calls the correspondence of the articulations and of the sanguineous system, we find many pertinent remarks; but we have not space for so minute an analysis, and must hasten to the conclusion of our labours: we shall, however, pause to extract one or two remarks relative to the habits and periods of disease. "It often happens (says our author,) that inflammation shall take place in an organ different from that which has been stimulated, and that in consequence of a disposition which is called *a habit.* Thus cold, moisture, &c. though acting upon the feet, produce sore throats, colics, pleurisies, and according to the susceptibility of the organ receiving the impression,—that is, according to the frequency with which such organ has been before diseased. (p. 380.)

Hemorrhages appear to depend occasionally upon this cause, and they also afford an example of what he calls *periodicité;* for they often recur, he observes, in the night, rather than in the day. Women frequently are attacked with the menstrual discharge during their sleep; others always miscarry at the third month. Many other diseases are also renewed about the same hour,—such as asthma, the hooping-cough, pains of various kinds. These instances will, we think, be sufficient to show the author's meaning, and enable the reflecting reader to follow the same train of thought.

We have now arrived at the fifth chapter, on the *formation and seat of maladies*, and of that vital phenomenon called *fever.* The first portion of this long chapter, though full of ingenuity, and in M. Foderé's best style, does not offer any thing that we consider of practical importance: nay, much of what he advances has been, and still continues to be, a matter of dispute. We shall merely insert the following illustration of the effects of stimulating applications overcoming diseases of irritation under certain circumstances,—such as the cure of gonorrhœa by cubebs or balsam copaiba, in large doses; both medicines that have a truly stimulating effect on the urinary organs. Thus rum, hot wine, &c. are often successfully employed by the vulgar in the commencement of colds and affections of the chest: yet, says our author, it is not fair to say that these diseases have been *cured* by these means,—they have only been *prevented: the patient has played at the game of double or quit.*

Fever is said to be merely a natural consequence of vital reaction against its causes, and in the train of phenomena he considers the nervous system as the first mover: for, if the brain is oppressed, this phenomenon does not take place, and, instead of the circulation being quickened, it becomes slower. This leads to a consideration of what the French have lately made the subject of great discussion, the existence of *essential* fever, unconnected with or unaccompanied by any local affection whatever. The Medical Society of Paris offered this as a prize question in 1819: nine candidates presented theses on the subject, but it could not be adjudged, and the same question was again offered as the subject for the prize of 1821. To us it appears something like the dispute as to the colour of the chameleon: it will vary according to the taste and talent of the observer; and it may be more than doubted whether fever ever runs its course to the death of the patient without producing some appearances of unequivocal disease; and, in the instance of the brain, for example, who shall say that all its morbid appearances are either understood, or have even been the subject of observation? In the event of the recovery of the patient, the dispute is evidently incapable of a decision.

Our author, declining to give a definition of fever, asks the following question,—Is fever always an instrument of the *vis medicatrix naturæ?* a position which he doubts, and we think very wisely: nor does he see any resemblance between this affection and those *perturbations* which physicians often seek to excite for the relief of many diseases. Nevertheless, he considers that it may be useful occasionally; but the examples he adduces are at least but equivocal.

Chapter 6th, on the *general therapeutics of epidemic diseases.*—Although nature may be able to throw off some diseases, yet is the interference of the physician no less necessary in the greater number; and always in the instance of maladies arising from local causes,—such as a grain of sand in the eye, worms in the intestines, the strangulation of a hernia, &c.

In epidemic diseases, says our author, we have a simple and familiar example of these local causes, which are dissipated by local remedies: gastric fevers, occasioned by the bad qualities of food or drinks; the malignant pustular carbuncle, and which a prompt application of the cautery destroys; primitive inflammations of the viscera, which immediate bleeding puts an end to, before fever has become established. We need not pursue this subject further.

Our author next passes in review the principal therapeutical agents which we are in possession of,—such as bleeding, emetics, purgatives, bark (the specific of intermittent diseases), &c. &c.; but, before he mentions these agents, he speaks of sudorifics, since, he says, that nature points out sweating as a mode to be employed to throw off disease; but of which he can give no better example than that of one of the French commissioners at Barcelona, who declares that he escaped the yellow fever by that means only. We omit our author's observations on this subject, for they do not appear to us remarkable, either for their novelty or utility.

With regard to bleeding, M. Foderé boldly avows his regard for general bleeding, rather than the application of leeches, and so far we agree with him; but why he should draw any comparison between bleeding and opium, (the first of which, he says, is a much better sedative than the latter,) we are quite at a loss to divine: on this side of the water, at least, practitioners in general are not in need of such information; neither are they in the habit of ordering indifferently one or other of these remedies, to produce the same effect. We are not quite convinced, either, of our author's correct distinction between an inflammation which governs a fever, or a fever which governs an inflammation: for example, says he, "there is an essential difference between an hepatic fever and hepatitis,—between a peripneumonic fever and peripneumony,—between a phrenitic fever and phrensy, &c. To illustrate this distinction, he goes on to observe, that, when the inflammation is primitive, the pain and the other symptoms remain the same during the remission of the fever; but, when it is only secondary, the local symptoms augment and diminish with those of general fever; the pulse is weaker, and not so full. This is all very true; but no one dreams, we conceive, of calling these latter affections inflammations; though it by no means follows as a consequence, that, when a local pain is very violent, although but a *symptom*, that a bleeding may not be a very useful remedy.

On the subject of the administration of emetics, we need not repeat what our author has said, since the *new* doctrines are not likely to gain many advocates here; yet we cannot refrain from praising the general good sense and justice of his remarks. We are not, however, equally pleased with his observations on purgatives, though he has not carried his condemnation of these remedies quite to the same extent as the majority of his countrymen.

We shall pass over the remaining part of this chapter, merely observing, that the praises which M. Foderé bestows upon the specific virtues of the bark in remittent and intermittent fever, are, in our estimation, not warranted by facts. Indeed, in many cases of intermittents especially, we view the speedy and copious introduction of this medicine as a great evil, often smothering merely the symptoms of the disease, and laying the foundation for permanent visceral obstruction. Let those who practised in Walcheren say what was the result of the free administration of bark in the disease of that climate; and let the practitioner well consider and reflect upon what peculiar derangement of the stomach, liver, spleen, or other viscus, the phenomenon called ague is depending; let him set to work to get rid of that morbid state, and a very little bark will be necessary for the re-establishment of his patient. We should not, however, do our author justice, if we did not remark that the propriety of what we have said above is fully admitted by him, in reference to those fevers met with on the borders of the Mincio, the Oglio, the marsh of Berra, &c.

Chapter 7th, on *predisposition*, commences with some examples of the particular exemptions of individuals or nations in certain epidemics, and which, as M. Foderé observes, differ from poisons, as these destroy all the beings exposed to them; whereas, epidemics only attack those predisposed to receive them. This *individuality*, as our author terms it, refers to the age, sex, temperament, hereditary disposition, degree of civilization, climate, and food of those exposed to the causes of disease. With respect to differences of sex, it is universally known that women are less subject to malignant fevers than men; and it appears that, in the fever of Cadiz of 1804, out of 7387 deaths, 5810 were of the male sex. The same calamity showed also that, of one hundred sick of each sex, the greatest number of males who died were from twenty-one to forty years of age; of the females, between one and ten. The other points contained in this chapter are considered seriatim. M. Foderé displays much reading, and an intimate acquaintance with his subject, but there is nothing particularly striking that would induce us to make any additional extracts.

We have now reached the last section of M. Foderé's work, and have consequently only two chapters to consider: they are short. The first treats of *simple gastric fever*, and which, in fact, is merely the fruit of irritation affecting the stomach and primæ viæ; and in which, as our author remarks, the crisis is in the hands of the physician, and must not be left to nature. It may be complicated, he says, either with intermittent, contagious, or inflammatory fever; but then, according to our own notion, it is no longer gastric fever; and we cannot see the necessity for adding to the already-difficult subject of fever by so many divisions and subdivisions, and such endless refinement as we find in all foreign systematic works, and in some of our own too. This chapter terminates with the history of a medical student, who applied to M. Foderé with the symptoms of simple fever arising from indigestion, and who, rejecting the common remedies recommended by our author, and full of the doctrines of Broussais, had applied upwards of two hundred leeches to his stomach, as well as undergoing three bleedings from the arm, without the knowledge of his physician, and who died six days after he had ceased to visit him.

The second chapter is on *gastric worm-fever*, a term which our author seems to have invented, not because this disease is one single and individual complaint, but because the complication of worms with fever augments the danger, and well deserves, in his opinion, a separate consideration. His observations here only apply to the presence of one species (the *lumbricus teres*), and his object is to present those diagnostic signs by which their presence may be known, whatever the form of the fever may be. We have read his list of symptoms over carefully, but we must confess that there is nothing in the whole detail that can afford a better diagnosis than we already possessed. The assemblage of all the signs he has enumerated will certainly lead the instructed physician to suspect the existence of worms; but, excepting their actual expulsion, these signs are all occasionally fallacious. The general causes of this affection he considers to be humidity of the air, and poor nourishment. A soft fibre, a lymphatic temperament, and sedentary employment, are the most frequent predisposing causes. It is better, upon the whole, says our author, to consider worms as a cause than as a symptom of disease, because, by taking that view of the case, we may often get rid of the disease before the fever has become established.

We cannot say much in favour of M. Foderé's curative means: he has a great dread of drastic purgatives, and recommends rhubarb, ipecacuanha, and magnesia, together with what he calls *boissons delayantes*, composed of decoctions of chamomile, chicory, &c. The anthelmentics he most relies upon are the oil of turpentine, tincture of assafœtida, calomel, male fernroot, and the seeds of the artemesia santonica. The volume ends with a lamentation on the degeneracy of the age, called forth by the fact that, both at Strasbourg and Dijon, cities in which disorders arising from worms are very frequent, owing to the want of good water at one place, and to the humidity of the corn at the other, large sums of money have lately been devoted to the erection of theatres, which should have been applied to the building of granaries, or the construction of fountains.

We trust our readers will not think that we have devoted too much space to the consideration of one volume only of a large work. M. Foderé's reputation, and the situation he holds as professor at a large university, rendered it, we conceived, incumbent upon us to take the earliest notice possible of his work, and to give as extended an account of its contents as its aphoristic form, and our limited space, would permit. Should M. Foderé be offended with the freedom of our remarks in some instances, he may be assured that we have not the less respect for him on that account; but that, following our usual plan, from which neither favour nor affection shall induce us to deviate, we have given such an account of his work as our reading and information induce us to believe to be just.

---

